# Long Non-Coding RNAs and Complex Human Diseases

**DOI:** 10.3390/ijms140918790

**Published:** 2013-09-12

**Authors:** Jing Li, Zhenyu Xuan, Changning Liu

**Affiliations:** 1 Bioinformatics Research Group, Key Laboratory of Intelligent Information Processing, Advanced Computer Research Center, Institute of Computing Technology, Chinese Academy of Sciences, Beijing 100190, China; E-Mail: lij.thu@gmail.com; 2 Department of Molecular and Cell Biology, Center for Systems Biology, University of Texas at Dallas, 800 W Campbell Road, Richardson, TX 75080, USA

**Keywords:** non-coding RNA, long non-coding RNA, complex human disease

## Abstract

Long non-coding RNAs (lncRNAs) are a heterogeneous class of RNAs that are generally defined as non-protein-coding transcripts longer than 200 nucleotides. Recently, an increasing number of studies have shown that lncRNAs can be involved in various critical biological processes, such as chromatin remodeling, gene transcription, and protein transport and trafficking. Moreover, lncRNAs are dysregulated in a number of complex human diseases, including coronary artery diseases, autoimmune diseases, neurological disorders, and various cancers, which indicates their important roles in these diseases. Here, we reviewed the current understanding of lncRNAs, including their definition and subclassification, regulatory functions, and potential roles in different types of complex human diseases.

## 1. Introduction

While about 20,000 protein-coding genes, representing less than 2% of the human genome, have been reported [1,2], a large part of the genome can be transcribed into non-coding RNAs (ncRNAs), which have little or no protein-coding capability [3,4]. Besides many widely studied classes of short ncRNAs, such as microRNAs (miRNA) and Piwi-interacting RNAs (piRNA) [5,6], one class of heterogeneous ncRNAs with lengths longer than 200 nucleotides, recently designated as long non-coding RNAs (lncRNAs), is increasingly attracting the attention of ncRNA researchers [7,8].

Over the past decade, furthered by the rapid progress in high-throughput sequencing technology, thousands of lncRNAs have been identified in mammalian transcriptomes [9,10]. A number of studies have revealed that lncRNAs can participate in various critical biological processes, such as chromatin remodeling, gene transcription, and protein transport and trafficking [11,12], implicating their impact on a wide range of complex human diseases [13,14]. However, despite some well-characterized lncRNAs, such as Xist and HOTAIR [15,16], little is known about the general features of most lncRNAs, such as gene structure, transcriptional regulation, and functional domains, and even less is known about their possible molecular mechanisms in different human diseases. Understanding the function of lncRNAs remains a significant challenge. Here, we review the current literature reporting on lncRNAs, including their definition, subclassification, regulatory functions, and roles in different types of complex human diseases.

## 2. Long Non-Coding RNAs: A Heterogeneous Class of RNAs

Long non-coding RNAs are most commonly defined as an RNA transcript more than 200 nucleotides (nt) long that cannot be translated into a protein [17]. However, this length threshold is not strict; it may vary 100 to 200 nt, or even longer. In different biochemical fractionation protocols, this threshold is primarily used to exclude most of the categories of small RNAs, such as small nucleolar RNA (snoRNA), microRNA (miRNA), Piwi-interacting RNA (piRNA), transfer RNA (tRNA), and small nuclear RNA (snRNA). Therefore, according to these simple criteria of transcript size and protein-coding capability, the designated lncRNAs contain a group of structurally and functionally heterogeneous RNAs, having a length that varies from approximately 200 nt to over 100 kb. They can undergo either splicing or not, with cellular locations in either nucleus or cytoplasm. They can be transcribed by RNA polymerase II or III, and they can play different functional and structural roles in different biological processes [18]. Based on these features, lncRNAs can be further categorized into different subgroups, as listed in Table 1. In past years, along with in-depth studies of lncRNAs, several lncRNA databases have been constructed (Table 2). These databases can facilitate further functional research on lncRNAs.

Genome-wide transcriptional maps have shown that lncRNAs are pervasively transcribed throughout mammalian genomes [39,40]. Clusters of overlapping sense and antisense transcripts can be found inside of known genes, as well as in intergenic regions. Natural antisense transcripts (NATs), which have been largely discovered in human, mouse, and many other species, are endogenous RNA molecules that exhibit partial or complete complementarities to other transcripts [22–24]. NATs may regulate the expression level of their sense counterparts. Several plausible regulation models, like blocking translation by sense-antisense pairing or antisense RNA-directed chromatin remodeling, have been proposed [13,41]. However, little mechanistic information has supported these suppositions, and more intensive studies are needed. Unlike NATs, long intergenic non-coding RNAs (lincRNAs) are large multiexonic RNAs, which are transcribed from intergenic regions, and may act in trans within large ribonucleoprotein complexes [10,19,42]. For example, Huarte *et al*. have reported that lincRNA-p21, which can physically interact with hnRNP-K, serves as a repressor in p53-dependent transcriptional responses by modulating hnRNP-K localization to chromatin [43]. A recent comprehensive screen has identified dozens of lincRNAs, which bind to multiple chromatin regulatory proteins, to affect related gene expression procedures and function critically in the pathway controlling pluripotent embryonic stem cell (ES) state [20]. Long non-coding RNAs have also been transcribed from widespread repetitive elements. For instance, human Alu and mouse B2 RNAs are originally derived from short interspersed repeat elements (SINEs). They are transcribed by RNA polymerase III in response to environmental stresses, such as heat shock, and act as transcriptional repressors by directly targeting RNA polymerase II [27–29]. Another interesting example is lncRNA PTENP1, a biologically active pseudogene of the tumor suppressor gene Phosphatase and Tensin Homolog (*PTEN*). PTENP1 performs a tumor suppressive function by acting as an “endogenous miRNA sponge”, which can positively regulate PTEN protein level via competing for PTEN-targeting miRNA binding [30,31].

Unlike protein-coding genes, which are usually conserved across species, most lncRNAs are poorly conserved and have been taken for transcriptional noise [44]. However, lack of conservation does not mean lack of function [45,46]. For example, two lncRNAs, Antisense Igf2r RNA (Air) and X Specific Transcript (Xist), are poorly conserved, but are well functional [47,48]. Their subjection to a series of recent and rapid adaptive selections may provide one explanation for the poor conservation of lncRNAs. For example, Highly Accelerated Region 1F (HAR1F), an lncRNA which is exclusively expressed in Cajal-Retzius neurons in the human neocortex, has undergone rapid evolutionary change in the human lineage since our last common ancestor of chimpanzee [49]. Moreover, for lncRNAs, which exert functions by secondary structures or short sequence motifs, we may only find small conserved regions interspersed in long poorly conserved transcripts [50]. Besides those poorly conserved lncRNAs, non-coding transcripts can also be transcribed from ultraconserved genomic regions (UCRs). UCRs were first discovered in the sequence comparison of mouse, rat, and human genomes [51]. They are genomic elements longer than 200 bp with 100% identity between orthologous regions in these three genomes [52,53]. Genomic variations in UCRs, such as single nucleotide polymorphisms (SNPs), have been reported to be associated with increased cancer risk [54]. Genome-wide expression profiling has revealed that a large fraction of UCRs are transcribed (transcribed-UCRs, T-UCRs) with significant alteration at both DNA and RNA levels in adult chronic lymphocytic leukemias, as well as colorectal and hepatocellular carcinomas [32,55]. Recent study in neuroblastoma has discovered the relevance between expression levels of specific T-UCRs and important clinical-genetic parameters, suggesting that T-UCRs may be used as signatures associated with cancer diagnosis, prognosis, and treatment [55].

As a heterogeneous class of RNAs, lncRNAs have been implicated in the regulation of a number of complicated biological processes. A study in human cell lines suggests that about 30% of lncRNAs are specifically expressed in the nucleus [25]. Many of them are involved in chromatin remodeling complexes and mediate genomic silencing [10]. One of the most well-known examples is the participation of lncRNAs in X-chromosome inactivation (XCI), a process by which one of the two copies of the X chromosome present in female mammals is inactivated [15]. During XCI, Inactive X Specific Transcript (Xist), a 17-kb lncRNA transcribed from the XCI center, will accumulate on the inactive X chromosome and recruit Polycomb complexes for subsequent epigenetic modifications. Its antisense counterpart, Tsix, which is another lncRNA specifically expressed from the other X chromosome, can also interact with Polycomb complexes and maintain the activity of X chromosome [48]. Apart from in *trans* regulation, some lncRNAs can directly regulate gene expression in *cis*. By using knockdown approaches and reporter assays, Orom *et al*. have discovered an enhancer-like effect for a set of lncRNAs in human cell lines [56]. Depletion of these lncRNAs leads to decreased expression of their neighboring protein-coding genes. More interestingly, a recent genome-wide study of transcriptional enhancers in mouse has shown that some lncRNAs, termed as enhancer-RNAs (eRNAs), are transcribed from functional enhancers [33]. Although the function of eRNAs remains largely unclear, their close correlation with active enhancers suggests an important role of eRNAs in transcriptional activation. Long non-coding RNAs are also actively involved in diverse cytoplasmic processes. One of the well-studied examples is noncoding repressor of *NFAT* (NRON), an lncRNA repressor of nuclear factor of activated T cells (*NFAT*). Using an arrayed library of short hairpin RNAs and cell-based assays, Willingham *et al*. have identified that NRON interacts with multiple proteins, including members of the importin-beta superfamily, and possibly functions as a specific regulator of *NFAT* nuclear trafficking [57]. Moreover, lncRNAs are probably involved in stress-related signaling pathways. Utilizing whole-genome tiling arrays, Silva *et al*. identified a new class of long stress responsive non-coding transcripts (LSINCTs), which have increased expression in response to DNA damage induced by the tobacco carcinogen 4-(methylnitrosamino)-1-(3-pyridyl)-1-butanone (NNK) [58]. Interestingly, LSINCTs also have increased expression in a number of cancer-derived cell lines, indicating its stress response under a carcinogenic environment.

## 3. Long Non-Coding RNAs and Complex Human Diseases

Complex diseases are multifactorial or polygenic disorders of the body. They are likely caused by multiple genetic variants with low penetrance in combination with various environmental and lifestyle factors, and they do not simply obey the standard Mendelian patterns of inheritance [59,60]. Coronary artery diseases, autoimmune diseases, neurological disorders, various cancers, and many other diseases all belong within this classification. The recent discovery that lncRNAs can participate in a wide range of biological processes has attracted substantial scientific interest in their potential impact on these complex diseases. It has been reported that lncRNAs are dysregulated in a variety of complex human diseases and are closely associated with disease development and progression (Table 3). Here, we describe some of the well-characterized lncRNAs associated with different types of complex human diseases.

### 3.1. LncRNAs in Coronary Artery Diseases

Facilitated by single nucleotide polymorphism (SNP) array, genome-wide association study (GWAS) is becoming one of the most powerful approaches to identify genetic variants susceptible to common diseases [117]. From these studies, a large amount of disease-associated SNPs are found to be mapped to non-coding genomic regions. While some of these SNPs could be associated with enhancers, it would not be surprising that many others are associated with lncRNAs. By using 52,608 haplotype-based SNP markers, the lncRNA called Myocardial Infarction Associated Transcript (MIAT) was first identified in a large-scale case-control association study of the samples from 3435 MI patients and 3774 controls [98]. MIAT dwells at a susceptible locus for myocardial infarction (MI) on chromosome 22q12.1. This study discovered six SNPs showing significant association with MI in this locus. The MIAT transcript is approximately 10 kb in length and has five exons. No translational product is encoded in MIAT based on *in vitro* translation assay, which indicates that it is an lncRNA. From *in vitro* functional analyses, MIAT transcription is increased by the minor variant of one SNP in exon 5. In contrast to the non-risk allele, the risk allele has more intense binding of nuclear protein(s). The study concluded that MIAT may play some roles in the pathogenesis of MI with altered expression by SNP [98].

Recent GWASs have identified a region on chromosome 9p that is associated with coronary artery disease (CAD) [118,119]. A long non-coding antisense RNA gene, named as Antisense non-coding RNA in the *INK4* locus (ANRIL), is a prime candidate for the chromosome 9p CAD locus [64]. Several recent GWASs showed that ANRIL has increased susceptibility to intracranial aneurysm, breast cancer, glioma, and basal cell carcinomas [65–68]. ANRIL is located in the *INK4b*/*ARF*/*INK4a* locus, and it is coregulated with *INK4a*, *INK4b* and *ARF*. Expression studies have confirmed that ANRIL is expressed in multiple atherosclerosis-related cell lines, including vascular endothelial cell, monocyte-derived macrophages and coronary smooth muscle cells [64,120]. Moreover, a mouse model study has demonstrated the pivotal role of ANRIL in the regulation of *INK4a/b* expression through a *cis*-acting mechanism and its implication in proliferation and senescence [121,122]. Interestingly, several studies discovered that Polycomb complexes are able to bind the *INK4*/*ARF* locus and alter expression of *INK4a* and *INK4b* [123,124]. Similar to lncRNA XIST, the ANRIL gene presents unusual masses of repetitive elements, as well as many binding sites for repressive transcription factors [120]. All these observations suggest that ANRIL, much like XIST, may regulate the expression of the INK4a/b transcript by recruiting Polycomb complex to the *INK4*/*ARF* locus and imposing a repressive chromatin state.

### 3.2. LncRNAs in Autoimmune Diseases

Long non-coding RNAs may also function in the regulation of downstream protein-coding genes, thus forming a complicated mutual regulation network with both coding and non-coding genes [125,126]. Recent studies have shown that autoimmune diseases, which result from an inappropriate immune response of the body against substances and tissues normally present in the body, have a complex genetic context that involves multiple protein-coding and non-coding genes. For example, in association study of 515 affected individuals and 526 controls, Shirasawa *et al*. discovered that the T allele of SNP Ex9b-SNP10 is correlated to increased risk for autoimmune thyroid disease (AITD) [106]. The Ex9b-SNP10 resides in intron 9 of the protein-coding gene *ZFAT* and the promoter region of an lncRNA, SAS-ZFAT, which is an antisense transcript of the ZFAT gene. With the existence of SNP Ex9b-SNP10, SAS-ZFAT expression is evidently upregulated, which, in turn, downregulates the expression level of its sense counterpart—truncated *ZFAT*. Since SAS-ZFAT is exclusively expressed in CD19+ B cells in peripheral blood lymphocytes, these results implicated that SAS-ZFAT might play a critical role in B cell function and determine susceptibility to AITD.

Another example is lncRNA PRINS, a Psoriasis Susceptibility-related non-coding RNA gene that harbors two Alu elements [103]. PRINS is transcribed by RNA polymerase II and is expressed at different levels in various human tissues. Real-time RT-PCR analysis showed that PRINS has higher expression in the uninvolved epidermis of psoriatic patients than in both psoriatic lesional and healthy epidermis, suggesting that PRINS plays a role in psoriasis susceptibility. *In silico* structural and homology studies have suggested that PRINS acts as a non-coding RNA. The RNA expression level of PRINS is decreased in the uninvolved psoriatic, but not healthy, epidermis with treatment of T-lymphokines that are known to precipitate psoriatic symptoms. Moreover, downregulating the RNA level of PRINS by RNAi can impair cell viability after serum starvation, but not under normal serum conditions. It was also discovered that PRINS could function as a “riboregulator” to regulate the expression of other genes involved in the proliferation and survival of cells.

### 3.3. LncRNAs in Neurological Disorders

Neurological disorders are diseases of the body’s nervous systems, which include the central nervous system, the peripheral nervous system, and the autonomic nervous system. Previous transcriptome studies have shown a number of lncRNAs in the mammalian brain, and most of them exhibit particular expression profiles within specific neuroanatomical regions, cell types, or subcellular compartments, implicating that lncRNAs probably have a significant impact on neurological regulation [127].

Long non-coding RNAs may participate in the pathogenesis of fragile X syndrome (FXS) and fragile X tremor ataxia syndrome (FXTAS), both of which are caused by the aberrant expansion of CGG trinucleotide, repeat in the 5′ UTR of protein-coding fragile-X mental retardation 1 gene (*FMR1*) [128,129]. Studies have shown that two lncRNAs, FMR4 and ASFMR1, are expressed from the *FMR1* locus. FMR4 is a primate-specific lncRNA that likely shares a bidirectional promoter with *FMR1* [79], while ASFMR1 is a spliced and poly-adenylated antisense transcript that overlaps the 5′ UTR CGG repeat region of *FMR1* [69]. *In vitro* studies of FMR4 have shown that it may function to prevent neurons or their progenitors from apoptosis during the progress of development in human. FMR4 and ASFMR1, as well as *FMR1*, may participate synergistically in neurological regulation in a RNA-protein interacting manner, since they are all silenced in FXS or upregulated in FXTAS patients. Dysregulation of these delicate interactions may result in various neurological disorders [69,79].

Long non-coding RNAs have also been reported to be dysregulated in different types of neurodegenerative diseases, such as spinocerebellar ataxia type 8 (SCA8) and Alzheimer’s disease (AD). SCA8 is an autosomal dominant disorder caused by repeat expansion [130]. Two bidirectionally transcribed genes are located within the SCA8 expansion region: the protein-coding gene ataxin 8 (*ATXN8*), with a CAG expansion that encodes a polyglutamine expansion tract protein, and ataxin 8 opposite strand (ATXN8OS) which is an lncRNA with a CUG repeat. In studies of transgenic mice expressing SCA8 expansion, it was found that the ATXN8OS mutant is overexpressed and co-localized with Muscleblind-like splicing regulator 1 (*MBNL1*) in neurons, which can lead to dysregulation of MBNL1-mediated alternative splicing, loss of GABAergic inhibition within the granular cell layer, and set the stage for the occurrence of disease [70]. AD is a form of dementia, which is believed to be caused by the formation of amyloid plaques in neurons [131]. Studies have shown that BACE1-AS, an antisense lncRNA counterpart of protein-coding gene *BACE1*, is highly expressed in tissues from AD patients [71]. *BACE1* is an enzyme that is responsible for amyloid precursor protein (APP) cleavage into amyloid β peptides, which form amyloid plaques in the neurons of AD patients [132]. Upregulation of BACE1-AS promotes the stabilization of *BACE1* mRNA and boosts the expression of *BACE1* protein, which leads to the production of pathogenic amyloid β peptides and thus may speed up the pathogenesis of AD [71]. Another lncRNA involved in AD is BC200, which is expressed almost exclusively in neuronal cells. In AD patients, the level of BC200 becomes upregulated. The increased expression of BC200 was found to be correlated with the severity of AD [74]. In addition, studies of BC1, the mouse functional homolog of BC200, have shown that BC1 knockout mice exhibit behavioral changes, thus demonstrating an important role for BC1 in brain function [133]. All these results suggest that dysregulation of BC200 may contribute to AD susceptibility.

### 3.4. LncRNAs in Cancers

Cancer is a broad group of various diseases in which abnormal cells divide uncontrollably and tend to invade other tissues. Up to now, although hundreds of oncogenes and tumor suppressor genes have been identified, the exact cause of most cancers remains unknown or poorly understood. In recent years, researchers have increasingly come to recognize lncRNAs as major mediators in cancer pathogenesis [134]. Thus far, no concrete evidence has surfaced to indicate any lncRNAs as causal factors in cancer. However, many lncRNAs have been found to be differentially expressed in a variety of cancers and may act as either oncogenes, such as MALAT-1, HOTAIR, and ANRIL, or tumor suppressor genes, such as MEG3, lincRNA-p21, and PTENP1, in cancer development. Here, we discuss some examples of such lncRNAs.

Like protein-coding oncogenes, some lncRNAs can promote cell proliferation and induce tumorigenesis. Metastasis-associated lung adenocarcinoma transcript 1 (MALAT-1), which correlates with high metastasis and poor prognosis in non-small-cell lung cancer, is an abundant 8.7-kb lncRNA encoded in the human chromosome 11q13 [93]. MALAT-1 is broadly expressed in normal human tissues and is found to be upregulated in many solid tumors, such as lung, breast, prostate, liver, and colon tumors [91,92,135]. MALAT-1 is believed to play a vital role in cell proliferation, migration, and invasion. By interacting with serine-arginine-rich splicing factor (*SR*), which is responsible for alternative splicing (AS) in a concentration- and phosphorylation-dependent manner, studies have shown that MALAT-1 can modulate the phosphorylation of *SR* proteins and thus regulate AS of selective pre-mRNAs [136]. MALAT1 is also involved in the regulation of cell mobility. RNAi-mediated silencing of MALAT1 impaired the *in vitro* migration of lung adenocarcinoma cells and reduced cell proliferation and invasive potential in a cervical cancer cell line [92]. Another onco-lncRNA example is *HOX* Antisense Intergenic RNA (HOTAIR). HOTAIR, a 2.2-kb spliced and poly-adenylated lncRNA, is transcribed from the antisense strand of the Homeobox C (*HOXC*) gene cluster on chromosome 12 [16]. Studies have shown that *HOTAIR* is overexpressed in breast tumors, hepatocellular carcinoma, and colorectal cancer [137–139]. A high level of HOTAIR expression is directly correlated with poor patient prognosis and metastasis. Recent studies revealed that HOTAIR is likely to work as a molecular scaffold to bind two distinct histone modification complexes, the Polycomb repressor complex 2 (PRC2) and the histone demethylase LSD1, facilitating their genome-wide retargeting to specific regions for coupled histone H3K27 methylation and H3K4 demethylation [140]. *In vitro* studies have shown that overexpression of HOTAIR in cell lines leads to the recruitment of PRC2 and LSD1 to over 800 additional loci, including those of tumor suppressor genes [16]. These observations indicate that dysregulation of HOTAIR may reprogram the epigenetic information to promote tumor cell invasion and subsequent metastasis.

Long non-coding RNAs can also act as tumor suppressor genes. One example is maternally expressed gene 3 (*MEG3*), a maternally imprinted RNA gene of approximately 1700 nucleotides [94]. Studies have revealed that *MEG3* is expressed in many normal tissues, but not in the majority of human meningiomas or human meningioma cell lines [141]. Moreover, ectopic expression of *MEG3* was found to suppress the growth of several human cancer cell lines, further supporting the effect of *MEG3* on tumor suppression [95]. MEG3 was found to be a positive regulator of *p53*, a tumor suppressor protein [142]. In cells that are transfected with MEG3, *p53* protein level increases significantly, which results in dramatically stimulating the transcription of p53-dependent genes from a p53-responsive promoter. Studies have shown that MEG3 is also capable of inhibiting cell proliferation in the absence of *p53* [143]. These data suggest that MEG3 can function as a tumor suppressor through both p53-dependent and p53-independent pathways. MEG3 has a total of twelve isoforms from alternative splicing, all of which contain three distinct secondary folding motifs (M1, M2, and M3). Deletion analysis indicates that motifs M2 and M3 are important for *p53* activation. Furthermore, a hybrid MEG3 RNA, which contains a piece of unrelated sequence, but preserves the original secondary structure, retained the functions of both *p53* activation and growth suppression [144]. As a regulatory lncRNA, all of these experiments demonstrated that the proper conformation of MEG3 is critical to its biological functions.

## 4. Conclusions

Long non-coding RNAs are rapidly becoming a focal point for intensified research in the biological and medical sciences. Increasing evidence has indicated that lncRNAs play important roles in various critical biological processes and that they add a new layer of complexity to already complex human diseases. We believe that the further functional and mechanistic studies of these versatile macromolecules will expand our understanding of general principles in biological systems and provide new approaches to the diagnosis and treatment of complex human diseases.

## Figures and Tables

**Table 1:** Types of long non-coding RNAs.

Long non-coding RNA	Symbol	References
Long intergenic non-coding RNA	LincRNA	[19,20]
Long intronic non-coding RNA		[14,21]
Natural antisense transcript	NAT	[22–24]
Promoter-associated long RNA	PALR	[25]
Promoter upstream transcript	PROMPT	[26]
Repetitive element-associated non-coding RNA		[27–29]
Transcribed pseudogene		[30,31]
Transcribed ultraconserved region	T-UCR	[32]
Enhancer-like non-coding RNA	eRNA	[33]

**Table 2:** Public lncRNA databases.

Name	Website	Reference
ChIPBase	http://deepbase.sysu.edu.cn/chipbase/	[34]
fRNAdb	http://www.ncrna.org/frnadb/	[35]
LNCipedia	http://www.lncipedia.org/	[36]
lncRNAdb	http://www.lncrnadb.org/	[37]
NONCODE	http://www.noncode.org/	[9]
NRED	http://nred.matticklab.com/cgi-bin/ncrnadb.pl	[38]

**Table 3:** Examples of lncRNAs dysregulated in complex human diseases.

LncRNA	Disease	References
aHIF	Multiple cancers	[61,62]
AK023948	Papillary thyroid carcinoma	[63]
ANRIL	Coronary artery disease; Multiple cancers	[64–68]
ASFMR1	Fragile X syndrome; Fragile X tremor ataxia syndrome	[69]
ATXN8OS	Spinocerebellar ataxia type 8	[70]
BACE1-AS	Alzheimer’s disease	[71]
BC200	Alzheimer’s disease; Multiple cancers	[72–74]
BIC	B-cell lymphoma	[75]
CUDR	Squamous carcinoma	[76]
DD3	Prostate cancer	[77,78]
FMR4	Fragile X syndrome; Fragile X tremor ataxia syndrome	[79]
GAS5	Breast cancer	[80]
H19	Multiple cancers	[81–85]
HOTAIR	Multiple cancers	[16,86]
HULC	Multiple cancers	[87,88]
Kcnq1ot1	Colon cancer	[89]
Kras1p	Prostate cancer	[30]
Linc-p21	Lung cancer	[43]
LOC285194	Osteosarcoma	[90]
MALAT-1	Multiple cancers	[91–93]
MEG3	Multiple cancers	[94–97]
MIAT	Myocardial infarction	[98]
ncRAN	Neuroblastoma	[99,100]
NDM29	Neuroblastoma	[101]
PCGEM1	Prostate cancer	[102]
PRINS	Psoriasis	[103]
PRNCR1	Prostate cancer	[104]
PTENP1	Prostate cancer	[30]
RMRP	Leukemia and lymphoma	[105]
SAS-ZFAT	Autoimmune thyroid disease	[106]
SPRY4-IT1	Melanoma	[107]
SRA-1	Breast cancer	[108,109]
TERC	Multiple cancers	[110]
Ube3a-as	Angelman syndrome	[111,112]
uc.73A	Colon cancer	[32]
UCA1	Bladder cancer	[113–115]
Zfas1	Breast cancer	[116]
